# Beyond Feature Selection: Interpretable Machine Learning for Mechanistic Insights in Metabolomics

**DOI:** 10.3390/biology15070572

**Published:** 2026-04-02

**Authors:** Haotian Bai, Yufei Ren, Jihan Wang, Yangyang Wang, Yanning Yang

**Affiliations:** 1 School of Physics and Electronic Information, Yan’an University, Yan’an 716000, China; 2 Yan’an Medical College, Yan’an University, Yan’an 716000, China

**Keywords:** interpretable machine learning, metabolomics, biomarker discovery, SHAP, biological insight, precision medicine

## Abstract

While metabolomics captures the dynamic chemical landscape of biological systems, its inherent high dimensionality and complexity pose significant analytical hurdles. Interpretable Machine Learning (IML) is revolutionizing the field by moving beyond traditional feature selection to extract biologically meaningful insights alongside robust predictions. This review systematically examines IML’s application in metabolomic biomarker discovery. We highlight how interpretation frameworks decode the key metabolites driving model decisions, transforming opaque “black-box” algorithms into testable mechanistic hypotheses. By evaluating cutting-edge studies across various pathologies, we illustrate IML’s pivotal role in disease subtyping, early diagnosis, treatment prediction, and mitigating demographic disparities. Although challenges in data generalizability persist, IML remains an indispensable bridge between computational prediction and biological understanding, ultimately advancing precision medicine.

## Simple Summary

Modern medicine increasingly analyzes thousands of small chemicals in the human body to detect diseases. However, making sense of this massive data using standard artificial intelligence often feels like using a “black box,” where computers provide medical predictions without explaining their reasoning. This review explores a new approach called interpretable machine learning, which forces computers to show their work. We discuss how these transparent computers models help scientists confidently identify specific chemical warning signs for complex illnesses, such as cancer and brain disorders. Furthermore, these transparent tools help researchers understand the actual biological causes of diseases and prevent biased medical results based on a patient’s age or gender. Ultimately, by bridging the gap between computer predictions and human understanding, transparent artificial intelligence will help doctors diagnose diseases earlier, discover safer drugs, and provide truly personalized healthcare for everyone.

## 1. Introduction

Metabolomics represents the functional endpoint of the omics cascade and is pivotal for biomarker discovery [1]. However, clinical translation is impeded by the inherent high dimensionality (n ≪ p) of metabolomic datasets [2]. Conventional linear models, such as PCA and PLS-DA, frequently fail to capture complex non-linear biological interactions [3,4]. Furthermore, standard feature selection often prioritizes correlated surrogates over causal drivers [5]. Consequently, this reliance on correlation yields spurious biomarkers lacking mechanistic plausibility, thereby compromising reproducibility and external validation [5,6].

To address these complexity challenges, Machine Learning (ML) algorithms—such as Random Forests (RF), Support Vector Machines (SVM), and Deep Learning (DL)—have been increasingly adopted for their superior predictive performance and ability to model non-linear metabolic landscapes [7,8]. Despite their high accuracy, complex ML models often operate as “black boxes”, where the internal logic driving predictions remains opaque [6]. In the biomedical domain, a “black box” is problematic; clinicians and biologists require not only an accurate prediction (e.g., disease diagnosis) but also an understanding of why a specific decision was made [6,9]. The lack of transparency hinders the discovery of underlying biological mechanisms and raises concerns regarding trust and safety in clinical decision-support systems [10]. Consequently, there is an urgent need to shift from purely predictive modeling to explainable frameworks that bridge the gap between computational power and biological interpretation [11].

IML has emerged as a pivotal framework to resolve this “accuracy–interpretability” dilemma [12]. IML methodologies generally fall into two paradigms: intrinsically interpretable models (e.g., linear regression, decision trees) and post hoc explanation methods [6]. While intrinsically interpretable models offer transparency by design, their structural simplicity often restricts their capacity to capture the stochastic and highly non-linear interactions characteristic of metabolic networks, thereby limiting predictive sensitivity. Consequently, post hoc model-agnostic methods have gained prominence as a superior strategy for metabolomics; they retain the predictive supremacy of “black-box” architectures while retroactively extracting transparent insights [12]. Crucially, moving beyond traditional feature selection that merely outputs a static ranking of variable importance (e.g., VIP scores), these post hoc techniques—exemplified by SHapley Additive exPlanations (SHAP)—provide granular, multidimensional biological insights. They enable the quantification of directionality (positive vs. negative impact), the visualization of non-linear dose–response relationships, and the identification of synergistic interactions between metabolic pathways [13]. Furthermore, by offering local interpretability, explaining predictions for individual samples, these methods facilitate the transition from population-level biomarker lists to patient-specific mechanisms required for precision medicine [11], an objective that, in the current scenario, is most effectively achieved through the integration of federated learning frameworks.

To clarify the computational inputs, metabolomic features vary by analytical approach. Targeted metabolomics typically utilizes quantified concentrations of known metabolites (e.g., amino acids or lipids). Conversely, untargeted metabolomics yields a vastly higher-dimensional feature space of instrumental variables, such as peak intensities, mass-to-charge ratios (*m*/*z*), and retention times (RT) in Mass Spectrometry (MS), or chemical shifts (ppm) in NMR. Finally, these features are frequently concatenated with clinical metadata (e.g., age, BMI) to form the complete feature matrix for IML training.

**Figure 1:**
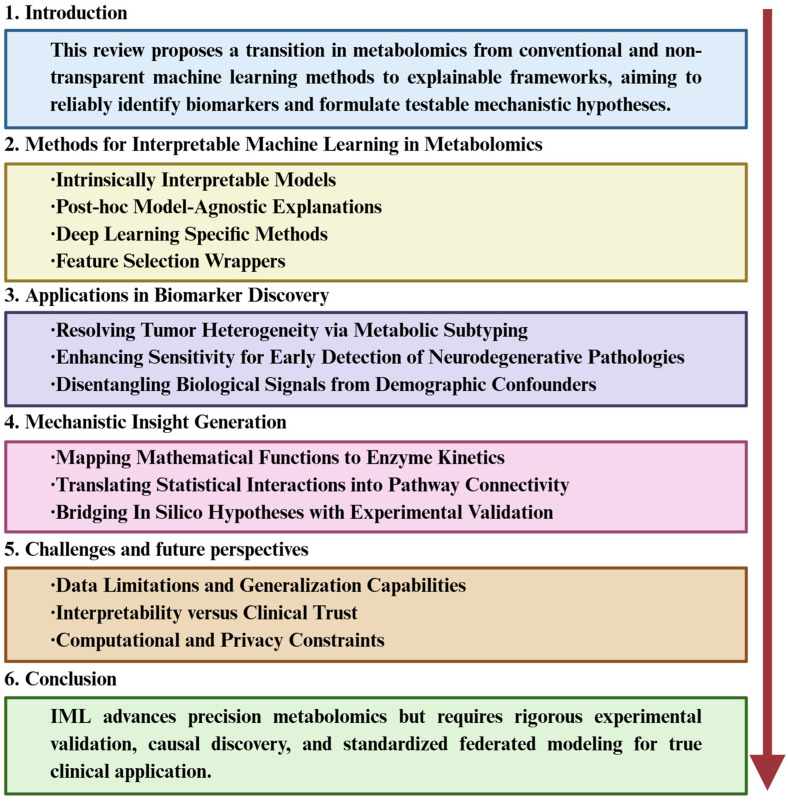
An overview of the organizational framework of this review. This framework first introduces the shift from conventional to explainable machine learning in metabolomics, outlines key interpretable methods, and details their applications in biomarker discovery and mechanistic insight generation. It then discusses current challenges and future directions, concluding with the role of IML in advancing transparent, clinically relevant research.

The primary objective of this review is to systematically examine the application of IML in metabolomics, advocating for a paradigm shift “beyond feature selection”. We aim to demonstrate how IML tools can be leveraged to not only identify robust biomarkers but also generate testable mechanistic hypotheses. The organizational framework of this review is presented in Figure 1. The remainder of this article is organized as follows: Section 2 details key IML methodologies for metabolomic data; Section 3 reviews cutting-edge applications in disease contexts, including tumor subtyping, early neurodegenerative detection, and confounder disentanglement; Section 4 discusses mechanistic insight generation from model explanations; and finally, Section 5 and Section 6 address current challenges and future directions for integrating IML into causal, multi-omics-driven precision medicine workflows.

## 2. Methods for Interpretable Machine Learning in Metabolomics

The inherent complexity and high dimensionality of metabolomics data necessitate analytical approaches that offer transparency alongside predictive accuracy [1,2]. IML provides a diverse toolkit to address this challenge, ranging from models with intrinsic transparency to post hoc techniques that elucidate opaque “black-box” algorithms [14,15]. This section categorizes these methodologies into four distinct classes: intrinsically interpretable models, post hoc model-agnostic explanations, specialized methods for deep learning, and feature selection wrappers [4,15,16].

### 2.1. Intrinsically Interpretable Models

Intrinsically interpretable models, characterized by parsimonious architectures where internal parameters directly quantify feature importance, remain the foundational tools of metabolomic analysis [14]. Partial Least Squares-Discriminant Analysis (PLS-DA) is widely considered the gold standard [3], employing Variable Importance in Projection (VIP) scores to identify discriminatory biomarkers; however, it is limited by linearity assumptions and a propensity for overfitting if not rigorously validated [1]. Similarly, regularized regression offers direct interpretability via coefficients (*β*), with Elastic Net (L1 + L2) often outperforming Lasso (L1) in metabolomics due to its superior handling of high multicollinearity [17,18]. Regarding non-linear approaches, single decision trees provide intuitive visualizations of decision paths via Gini impurity reduction, though they typically suffer from instability and lower predictive accuracy compared to ensemble methods [19]. Finally, Generalized Additive Models (GAMs) extend linear frameworks to capture complex biological patterns—such as U-shaped dose–response curves—through shape functions without compromising additive interpretability [20,21].

### 2.2. Post Hoc Model-Agnostic Explanations

To interpret complex “black-box” algorithms such as Random Forests, XGBoost, and SVMs, post hoc model-agnostic methods have become the standard in modern IML [15]. Among these, SHAP (SHapley Additive exPlanations) represents the state-of-the-art framework. Grounded in cooperative game theory, SHAP treats biological features as “players” in a coalition to fairly distribute the “payout” (the model’s prediction). By computing the average marginal contribution of a metabolite across all possible feature combinations, SHAP guarantees mathematical consistency and offers versatile insights—aggregating local SHAP values yields global importance rankings, while dependence plots reveal non-linear interactions between metabolites [11,20]. For local interpretability, LIME (Local Interpretable Model-agnostic Explanations) operates on the premise of local linearity. It approximates the decision boundary of a complex model by generating a synthetic dataset via random perturbation of a specific sample’s features. A simple, interpretable surrogate model (e.g., weighted linear regression) is then trained on this perturbed data to explain the individual prediction, although this reliance on random sampling can lead to instability in explanation consistency [6].

Beyond specific attribution frameworks, evaluating global feature behavior is critical. Permutation Feature Importance (PFI) provides a model-independent metric by measuring the degradation in predictive performance (e.g., accuracy or R2) after randomly shuffling the values of a single feature. While computationally efficient, PFI is susceptible to bias in metabolomics datasets characterized by high multicollinearity, as shuffling can create biologically impossible feature combinations [2]. Consequently, for visualizing marginal effects, Accumulated Local Effects (ALE) plots are often preferred over Partial Dependence Plots (PDP). Unlike PDPs, which assume feature independence, ALE calculates changes in prediction based on the conditional distribution of features, thereby robustly accounting for the confounding influence of co-regulated metabolic clusters [21].

### 2.3. Deep Learning Specific Methods

The integration of deep learning architectures—including DNNs, CNNs, and LSTMs—into metabolomics necessitates specialized attribution methods relying on gradients or backpropagation [22]. Layer-wise Relevance Propagation (LRP) facilitates this by retro-propagating relevance scores to identify key input features, such as specific spectral peaks [23]. Concurrently, Integrated Gradients (IG) mitigates the issue of gradient saturation by accumulating gradients relative to a baseline; this approach satisfies the axiom of completeness, thereby offering superior reliability compared to simple saliency maps [15,24]. Furthermore, for advanced architectures like Transformers and Graph Neural Networks (GNNs), attention mechanisms provide intrinsic interpretability by explicitly quantifying the weight assigned to specific metabolites or pathway nodes during the decision-making process [25,26].

### 2.4. Feature Selection Wrappers

**Figure 2:**
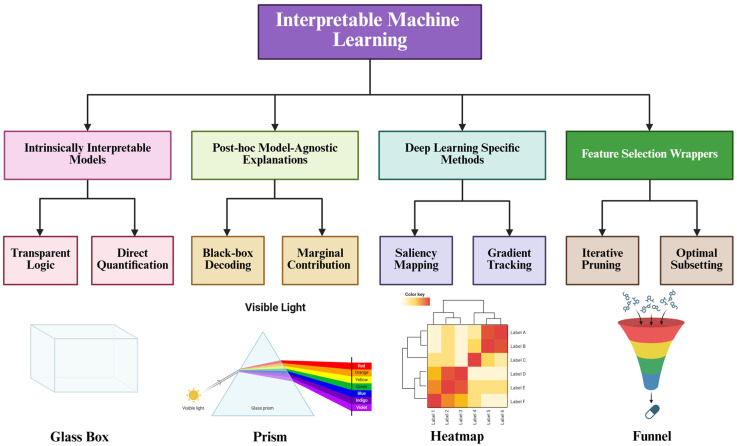
Taxonomy and conceptual mechanisms of IML methods in metabolomics. The upper panel illustrates a hierarchical classification of IML methodologies, categorizing them into four distinct classes: intrinsically interpretable models, post hoc model-agnostic explanations, deep learning-specific methods, and feature selection wrappers. The lower panel employs visual metaphors to elucidate the core operating principles of each category: (1) Glass Box: Represents intrinsically interpretable models, symbolizing architectures where the internal decision logic is fully transparent and parameters (e.g., coefficients) are directly accessible. (2) Prism: Symbolizes post hoc model-agnostic explanations (e.g., SHAP), which act to decompose a complex “black-box” prediction (white light) into individual marginal feature contributions (spectral colors). (3) Heatmap: Depicts deep learning-specific methods, utilizing gradient-based saliency or attention mechanisms to visualize relevant input features as “hotspots” within the network. (4) Funnel: Illustrates feature selection wrappers, visualizing the iterative filtration process that distills a large set of noisy metabolites into a concise, minimal-optimal biomarker subset.

Distinct from explanatory frameworks, wrapper methods are critical for identifying robust biomarker signatures through the rigorous evaluation of feature subsets. The Boruta algorithm, utilizing a Random Forest architecture, functions as an “all-relevant” feature selection method by comparing original features against randomized “shadow features” [27]. This approach is particularly valuable in metabolomics for preserving redundant yet biologically significant metabolites, such as co-regulated pathway members, thus facilitating comprehensive pathway analysis [28]. Conversely, Recursive Feature Elimination (RFE) employs an iterative process to prune the least informative features based on model weights. By isolating a “minimal-optimal” subset, RFE is ideally suited for developing clinical diagnostic panels where maximizing predictive performance with a concise biomarker panel is paramount [4,29]. Figure 2 illustrates the taxonomy and conceptual mechanisms of IML methods in metabolomics. Table 1 presents a comparison of interpretable machine learning methods in metabolomics.

## 3. Applications in Biomarker Discovery

In this section, we examine how IML bridges the gap between predictive opacity and clinical translation by transforming black-box outputs into biological hypotheses [14,46]. Specifically, we review its applications in elucidating metabolic heterogeneity for disease subtyping, enhancing sensitivity in early neurodegenerative detection, and disentangling biological signals from demographic confounders to ensure robust diagnostics [47].

### 3.1. Resolving Tumor Heterogeneity via Metabolic Subtyping

Cancer is not a monolithic disease but a heterogeneous spectrum of molecular pathologies [48]. Yet, conventional biomarker discovery often relies on reductionist case–control designs that treat populations as homogenous, masking critical subgroup-specific variations [49]. For instance, although the “Warburg effect” is a hallmark, metabolic reprogramming is highly context-dependent [50]; distinct subtypes may preferentially utilize glutaminolysis, fatty acid oxidation, or de novo lipid synthesis depending on oncogenic drivers (e.g., MYC vs. KRAS) and the microenvironment [51]. Consequently, failing to resolve this heterogeneity yields non-specific biomarkers, hindering personalized therapy and contributing to high clinical trial failure rates [52].

IML frameworks facilitate the transition from binary classification to sophisticated metabolic subtyping by elucidating the distinct feature dependencies that drive cluster formation [53]. Unlike traditional unsupervised clustering methods that rely solely on mathematical distance, IML-guided approaches—such as SHAP-enhanced clustering—explicitly identify the specific metabolic drivers characterizing each subtype [54]. To illustrate, XGBoost models coupled with SHAP analysis mechanistically differentiate breast cancer subtypes by visualizing lipid specificities. Rather than merely associating elevated phosphatidylcholines (PCs) with malignancy, SHAP summary plots explicitly reveal that PCs with longer acyl-chains and higher polyunsaturation yield high positive SHAP values, strongly driving the model output toward a Triple-Negative Breast Cancer (TNBC) prediction [55,56]. Conversely, shorter, saturated PCs exhibit negative SHAP values, favoring Luminal A diagnoses. This deep feature-level resolution clearly proves IML decodes complex lipidomic heterogeneity far beyond the limited capabilities of standard receptor status evaluations [48,56].

Crucially, IML facilitates the identification of context-specific biomarkers via interaction analysis, thereby capturing features—such as succinate in SDH-deficient tumors—that standard linear models frequently discard due to low global significance [56,57]. By utilizing algorithms like TreeExplainer to compute SHAP interaction values, researchers can quantify non-linear dependencies where the contribution of one metabolic feature is contingent upon another [11]. This approach enables the progression from single-marker hypotheses to “multi-hit” models; for instance, in colorectal cancer, IML has revealed that the predictive efficacy of plasma amino acids is modulated by the gut microbiome profile [58]. Ultimately, identifying these high-interaction pairs facilitates the construction of dynamic network models that reflect the plasticity of tumor metabolism, guiding the development of subtype-specific inhibitors that target specific vulnerabilities within the metabolic machinery [59].

### 3.2. Enhancing Sensitivity for Early Detection of Neurodegenerative Pathologies

Early detection of Alzheimer’s disease (AD) and Mild Cognitive Impairment (MCI) remains a formidable challenge in clinical metabolomics [60]. Peripheral metabolic signals in early neurodegeneration are typically subtle, non-linear, and obscured by homeostasis and the blood–brain barrier [61,62]. Conventional linear models, such as PLS-DA, often lack the sensitivity to distinguish these attenuated signals from biological noise [60]. As prodromal pathology involves complex network dysregulation rather than simple linear separation, these traditional methods frequently suffer from high false-negative rates, delaying diagnosis until irreversible neuronal damage occurs [60,63].

**Figure 3:**
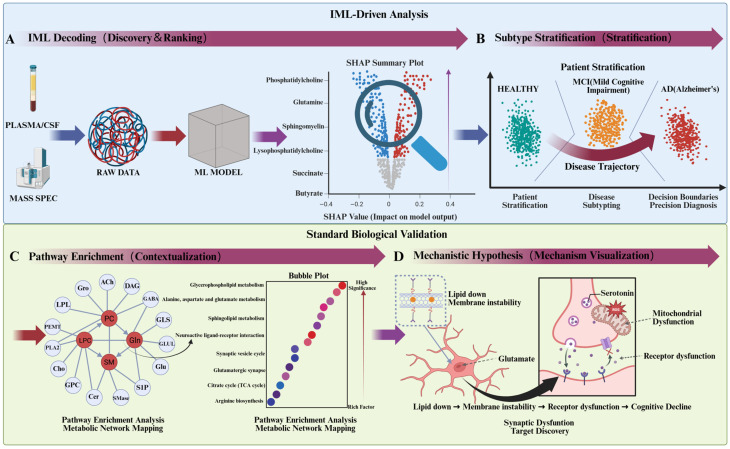
Integrated IML and biological validation workflow for biomarker discovery in Alzheimer’s disease (AD). (**A**) IML Decoding: High-dimensional plasma/CSF metabolomic data are processed via machine learning. Post hoc SHAP analysis prioritizes top biological drivers (e.g., PC, Gln, SM, LPC) based on their impact on model output. (**B**) Subtype Stratification: Utilizing these metabolic signatures, the model stratifies subjects into Healthy, Mild Cognitive Impairment (MCI), and AD clusters, defining precise diagnostic boundaries and continuous disease trajectories. (**C**) Pathway Enrichment: Key metabolites are mapped into an interaction network (left) revealing enzymatic relationships (e.g., PLA2, GLUL). A corresponding bubble plot (right) highlights significantly dysregulated pathways, prominently including glycerophospholipid/sphingolipid metabolism and glutamate signaling. (**D**) Mechanistic Hypothesis: IML insights are translated into a synaptic-level mechanism, linking metabolic alterations (e.g., lipid depletion) to downstream pathologies like mitochondrial ROS accumulation, altered neurotransmitter dynamics, and receptor dysfunction, ultimately precipitating cognitive decline.

IML empowers the detection of subtle metabolic signatures by modeling complex, non-linear interactions that elude conventional linear methods. While algorithms such as Random Forests, Support Vector Machines, and Deep Neural Networks exhibit superior capacity for handling high-dimensional metabolomic data, their utility in Alzheimer’s research is contingent upon interpretability. Advanced attribution techniques, such as Layer-wise Relevance Propagation (LRP) and Integrated Gradients, address this by projecting model decision logic back onto the input metabolome, thereby highlighting specific drivers of MCI such as kynurenine pathway alterations, distinct bile acid profiles, or lipid peroxidation products [61]. For instance, deep learning models can identify non-linear ratios between phosphatidylcholines (PC) and lysophosphatidylcholines (LPC) [63]; whereas univariate statistics often fail to detect differences in these lipids individually, IML reveals that their discordance is a key predictor of membrane instability in early neurodegeneration [63,64]. Ultimately, by defining such “combinatorial biomarkers,” IML provides a roadmap for targeted assays that prioritize pathway dysregulation over static metabolite abundance [65,66]. A comprehensive illustration of this IML-driven framework, demonstrating the progression from identifying raw metabolic signatures to generating testable mechanistic insights for Alzheimer’s disease, is presented in Figure 3.

Beyond identifying correlations, IML is instrumental in validating the mechanistic plausibility of peripheral biomarkers for central nervous system disorders [20], notably within gut–brain axis research linking microbiota-derived metabolites—such as short-chain fatty acids—to AD cognitive scores [67,68]. By utilizing SHAP dependence plots, researchers can delineate precise, non-linear concentration-risk profiles [41]; unlike linear correlation coefficients, these visualizations reveal complex threshold effects and biphasic (U-shaped) curves, indicating where metabolites transition from protective to deleterious levels [43]. This granularity is essential for distinguishing secondary compensatory responses from primary pathological drivers [69,70]. Consequently, by visualizing these decision boundaries, IML confirms the biological validity of identified biomarkers, facilitating the development of sensitive, mechanistically grounded blood-based screening tools [20,63].

### 3.3. Disentangling Biological Signals from Demographic Confounders

A pervasive challenge in biomarker discovery is the influence of confounding variables, as the human metabolome is acutely sensitive to non-disease phenotypic factors, including age, biological sex, BMI, and ethnicity [71]. In many datasets, putative disease biomarkers function merely as proxies for these demographic variances [72], precipitating the “Right for the Wrong Reasons” phenomenon [73]. For instance, algorithmic training on demographically unbalanced cohorts may result in models that predict age or ancestral background rather than pathological mechanisms [73,74]. Ultimately, this yields biomarkers that lack generalizability across diverse populations, thereby exacerbating health disparities and compromising diagnostic efficacy for underrepresented groups [75,76].

IML provides sophisticated frameworks to disentangle confounding effects and isolate genuine disease-associated signals. Visualization techniques, such as SHAP Dependence Plots and Accumulated Local Effects (ALE), facilitate the assessment of metabolite-outcome relationships while strictly controlling for covariates. This approach is particularly vital for mitigating racial disparities; by quantifying interaction effects between demographic variables and metabolite abundance, IML validates biomarker robustness across diverse ethnic groups [47]. Crucially, identifying features where predictive value relies on ancestral background—manifested as divergent risk function slopes in SHAP interaction analyses—allows researchers to detect and mitigate potential population specificity or algorithmic bias [11,47].

To systematically address these disparities, stratified interpretability analyses—such as Cohort-SHAP—can be employed to calculate feature importance across specific demographic strata [11,77]. This approach facilitates the identification of “invariant biomarkers” that maintain consistent predictive contributions regardless of race or gender, thereby isolating core biological pathologies [77], while simultaneously quantifying baseline shifts to inform population-adjusted clinical reference ranges [11]. Furthermore, while adversarial de-biasing strategies prevent models from predicting protected attributes [78,79], IML is not a permanent solution. A more attractive approach is federated modeling, which advances “Precision Health Equity” [78] by collaboratively training diagnostic algorithms across decentralized, diverse cohorts to permanently prevent the clinical deployment of biased AI tools. To illustrate the translational impact of these methodologies, Table 2 summarizes how diverse IML architectures extract disease-specific signatures, transcending traditional statistical limitations. These examples highlight how techniques like SHAP distill complex biological interactions into concise, cost-effective diagnostic panels, moving beyond static lists to elucidate phenomena like non-linear neuroinflammation responses and metabolic subtyping in oncology.

## 4. Mechanistic Insight Generation

Moving beyond the identification of diagnostic markers, the ultimate goal of metabolomics is to decode the etiology of disease. Although conventional feature selection identifies predictive metabolites, it fails to elucidate underlying biological interactions, necessitating a shift from static scalar metrics to the granular characterization of phenotypic trajectories [4,15]. IML frameworks address this limitation by employing sophisticated visualization tools—including Partial Dependence Plots, Accumulated Local Effects, and SHAP dependence plots—to reconstruct complex, non-linear metabolic topographies [11]. By delineating critical biological phenomena such as saturation points and hormetic responses that elude linear models, these techniques transform abstract algorithmic outputs into testable biochemical hypotheses, thereby elevating metabolomics from a descriptive task to a mechanistically driven discipline [22].

### 4.1. Mapping Mathematical Functions to Enzyme Kinetics

The inherent non-linearity of biological systems imposes severe constraints on traditional statistical methods, such as *t*-tests and logistic regression, which rely on assumptions of unbounded monotonicity [4]. Fundamentally, biological networks ensure metabolic flux is governed by enzymatic saturation, allosteric regulation, and feedback inhibition, resulting in complex, non-linear dose–response curves [48]. IML frameworks transcend these limitations by uncovering these dynamics without prior assumptions, mapping mathematical topographies directly to biological phenomena. Specifically, a frequent non-linear pattern elucidated by IML is the “saturation curve,” where metabolite risk contributions plateau beyond a specific threshold, mirroring Michaelis–Menten kinetics [87]. Crucially, data-driven IML architectures autonomously identify such biological “ceiling effects,” explicitly modeling the saturation of SGLT2 transporters in diabetes, where disease risk reliably stabilizes once precise physiological limits are breached [50,88]. Identifying these Vmax plateaus is critical for distinguishing rate-limiting steps from linear metabolic phases, thereby guiding targeted therapeutic strategies and generating hypotheses validatable through flux balance analysis [87,89].

Beyond saturation, linear models frequently fail to capture homeostasis, specifically the biphasic “Goldilocks effect” where metabolites exert protective functions within physiological ranges but become deleterious at extremes [90]. IML frameworks surmount this by revealing non-linear U-shaped relationships via SHAP dependence plots, where protective troughs are flanked by pathological deviations representing deficiency or toxicity. By mapping these inflection points, researchers can quantify precise homeostatic boundaries, such as those distinguishing malnutrition from lipotoxicity [91], thereby defining outcome-based reference intervals critical for characterizing diseases of metabolic dysregulation [11,92]. Closely related to homeostasis is hormesis, a biphasic dose–response phenomenon effectively elucidated by visualization tools like ALE plots [93,94].

These tools can distinguish protective Nrf2 pathway activation at low xenobiotic concentrations from cytotoxicity at high levels. Furthermore, IML detects biological “tipping points”—step-function discontinuities corresponding to phase transitions like mitochondrial collapse or inflammasome activation [95]. By pinpointing these thresholds, researchers can generate precise hypotheses regarding regulatory breakpoints to guide targeted experimental validation [95,96].

### 4.2. Translating Statistical Interactions into Pathway Connectivity

In contrast to the reductionist framework treating metabolites as orthogonal entities, biological systems are intrinsically interconnected, where phenotypes manifest as emergent properties of complex network perturbations [97,98]. While conventional linear models frequently discard these dependencies to mitigate multicollinearity, IML frameworks explicitly interrogate them to elucidate functional relationships [4]. By quantifying statistical interactions, wherein the predictive efficacy of one feature is modulated by another, researchers can reconstruct molecular crosstalk, mapping computational dependencies directly to physical pathway connectivity [11,15]. To elucidate these complex dependencies, IML transcends “Main Effects” by quantifying “Interaction Effects” via metrics like SHAP Interaction Values [11]. Biologically, these values serve as statistical signatures of biochemical synergy, antagonism, or dependency [15], allowing for the construction of “co-dependency networks” that delineate functional modules [15,22].

Biologically, strong statistical interactions often reflect upstream-downstream relationships, offering granular insights into metabolic flux dynamics that elude aggregate enrichment analyses [92]. For instance, within glycolysis, synergistic interactions between glucose and pyruvate typically indicate hyper-metabolic states (e.g., the Warburg effect), whereas discordant profiles signal rate-limiting bottlenecks or enzymatic inhibition [48,89]. By mapping these dependencies to canonical databases such as KEGG, IML frameworks facilitate the inference of reaction stoichiometry solely through the detection of deviations from expected substrate-product equilibria. Furthermore, metabolic homeostasis is contingent upon rigorous crosstalk between competing pathways. For instance, IML captures the Randle Cycle as a distinct negative interaction between plasma free fatty acids and glycolytic intermediates, modeling context-dependent pathogenicity [99,100]. Similarly, interactions between branched-chain amino acids and acylcarnitines serve as digital fingerprints for the interplay between protein catabolism and mitochondrial β-oxidation stress [101].

The elucidation of these interactions necessitates a paradigm shift from single-biomarker hypotheses to “multi-hit” models, particularly within complex pathologies where biological redundancy compromises the specificity of isolated metabolites. IML interaction analyses resolve this by identifying high-fidelity combinatorial biomarkers, such as the glutamine-glutamate ratio indicative of glutaminase activity, thereby capturing context-specific phenomena like synthetic lethality [102,103]. This approach aligns metabolomics with Network Medicine, reconceptualizing disease as the topological breakdown of functional modules rather than discrete node defects [104]. By reconstructing the dynamic architecture of metabolic networks, IML yields actionable mechanistic insights, suggesting that therapeutic strategies should target the uncoupling of pathological interactions or the restoration of regulatory feedback loops [59]. To facilitate the translation of these computational observations into biological hypotheses, Table 3 provides a systematic guide for decoding common IML visual patterns and their potential mechanistic correlates.

### 4.3. Bridging In Silico Hypotheses with Experimental Validation

**Figure 4:**
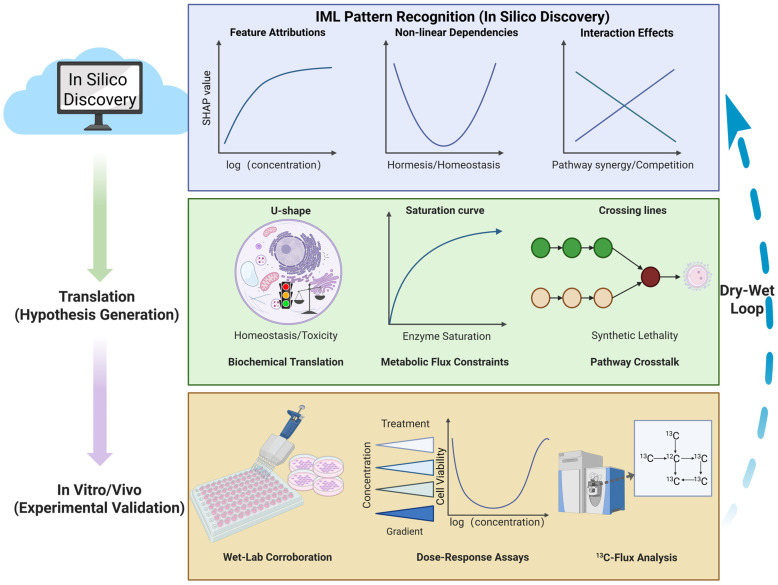
The “Dry-Wet” Loop framework: Bridging in silico IML patterns with experimental validation. The workflow is organized into three hierarchical tiers illustrating the translational pipeline: (**Top**) In Silico Discovery: IML algorithms identify abstract statistical patterns from high-dimensional metabolomics data, such as logarithmic saturation curves, non-linear hormetic (U-shaped) dependencies, and high-order interaction effects. (**Middle**) Hypothesis Generation: These mathematical topologies are translated into testable biochemical mechanisms. For instance, U-shaped patterns are mapped to mitochondrial homeostasis (balancing deficiency vs. toxicity), saturation curves to enzymatic kinetic limits, and crossing interactions to pathway crosstalk (e.g., synthetic lethality). (**Bottom**) Experimental Validation: Computational hypotheses are rigorously tested using orthogonal wet-lab techniques. High-throughput dose–response assays corroborate non-linear biological phenotypes, while stable isotope tracing (13C-Flux Analysis) confirms metabolic pathway alterations. The Dry-Wet Loop (right arrow) signifies the iterative refinement of computational models based on experimental ground truths.

The translational utility of computational metabolomics is predicated not merely on predictive metrics but fundamentally on the biological validity of the elucidated mechanisms [57]. Given that feature attributions derived from IML tools remain inherently correlative [6], transitioning from in silico hypotheses to bona fide discovery requires rigorous in vitro and in vivo corroboration [105]. This necessitates an iterative “Dry-Wet Loop” workflow, wherein computationally identified metabolic vulnerabilities are systematically interrogated using orthogonal wet-lab techniques. Figure 4 illustrates this translational workflow, demonstrating how abstract statistical patterns derived from IML (such as U-shaped curves or interaction effects) are translated into mechanistic hypotheses and subsequently verified through rigorous experimental assays like metabolic flux analysis.

A paramount application of this workflow is the validation of context-specific “synthetic lethal” interactions—such as those driving chemotherapy resistance—that frequently elude linear modeling [106]. Through SHAP interaction analysis, researchers have elucidated critical non-linear dependencies, such as the link between glutamine availability and SCD1 activity, predicting that tumor survival is contingent upon a simultaneous “multi-hit” mechanism [107,108]. Guided by these insights, targeted ^13^C-Metabolic Flux Analysis confirmed that resistant subtypes uniquely divert glutamine-derived carbon toward de novo lipogenesis to preserve membrane fluidity [109], converting statistical interactions into actionable therapeutic strategies [110].

**Table 3:** Decoding IML patterns: Translating computational features into biological mechanisms and validation strategies.

IML Visual Pattern (Observation)	Mathematical Interpretation	Putative Biological Mechanism	Specific Biological Examples	Suggested Wet-Lab Validation Strategy	References
Sigmoid/Plateau Curve (in SHAP/ALE Dependence Plot)	Risk contribution increases linearly then stabilizes after threshold X.	Enzyme Saturation (Vmax), Transporter Limitation, or Receptor Occupancy Saturation.	Glucose uptake via SGLT2 in diabetes; Folate uptake in cancer cells.	Metabolic Flux Analysis (MFA) with ^13^C-tracers to measure Vmax; Uptake Assays with radiolabeled substrates.	[41]
U-Shaped/J-Shaped Curve	Biphasic effect: Protective at physiological mean, pathogenic at extremes (deficiency/excess).	Homeostasis (“Goldilocks effect”), Hormesis, or Toxicity Thresholds.	Butyrate (SCFA) in gut–brain axis; ROS signaling vs. oxidative stress; Selenium or micronutrients.	Dose–Response Assays using a fine-grained concentration gradient (physiological to supraphysiological); Mitochondrial Respiration (Seahorse) assays.	[111]
Step-Function/Discontinuity	Sharp jump in risk score at a precise concentration point.	Biological Phase Transition (Tipping Point), Checkpoint Activation, or Membrane Collapse.	Mitochondrial Membrane Potential collapse leading to apoptosis; Inflammasome activation threshold.	Live-Cell Imaging to monitor real-time cellular events (e.g., Calcium flux, Apoptosis markers) around the predicted threshold.	[112]
High Positive Interaction (Synergistic Effect)	Risk (A + B) > Risk (A) + Risk (B)	Hyper-metabolic flux, Pathway Co-activation, or Positive Feedback Loop.	Glucose + Pyruvate in Warburg effect; Glutamine + Palmitate in ferroptosis resistance.	Dual-Knockdown/Inhibition of upstream enzymes; Isotope Tracing to confirm flux routing into the shared pathway.	[81]
High Negative/Discordant Interaction	Risk is highest when Metabolite A is High and B is Low (or vice versa).	Rate-limiting Bottleneck, Allosteric Inhibition (Crosstalk), or Synthetic Lethality.	Free Fatty Acids inhibiting Glucose oxidation (Randle Cycle); Succinate accumulation due to SDH defect.	Rescue Experiments (supplementing downstream metabolites); Enzyme Activity Assays to test for allosteric inhibition.	[20]
Cluster Importance (Group-SHAP)	A group of correlated features drives prediction collectively.	Pathway Dysregulation, Protein Complex Dysfunction, or Co-regulated Gene Expression.	TCA Cycle Intermediates collective downregulation; Lipid Class (e.g., Ceramides) elevation.	Multi-omics Integration (Proteomics/Transcriptomics) to validate pathway enrichment; Western blot for key pathway regulators.	[113]

Similarly, verifying the non-linear thresholds identified by IML—such as U-shaped hormetic relationships—requires rigorous dose–response experimentation [114,115]. By employing in vitro models treated with concentration gradients, researchers have confirmed biphasic physiological responses, validating that non-linearities captured by SHAP analysis represent authentic mechanisms rather than algorithmic overfitting [116,117,118]. Ultimately, bridging the divide between prediction and reality requires a diverse toolkit. Techniques such as stable isotope tracing distinguishes active flux from static accumulation [110,112], while genetic perturbations (CRISPR/Cas9) and proteomic analysis corroborate functional dependencies and enzyme status [119,120]. Integrating these orthogonal approaches with IML catalyzes a fundamental paradigm shift from “black-box” prediction to “white-box” discovery, ensuring that identified metabolic signatures are biologically authentic [92].

## 5. Challenges and Future Perspectives

### 5.1. Data Limitations and Generalization Capabilities

The primary impediment to robust IML in metabolomics is not merely dimensionality (n ≪ p), but the profound lack of standardized, multi-center datasets comparable to genomics’ TCGA. Current IML models are predominantly trained on single-center cohorts, making them highly susceptible to batch effects and instrument-specific noise (e.g., LC-MS drift) rather than capturing true biological signals. A critical analysis reveals that models achieving >90% accuracy in internal validation frequently fail in external cohorts due to “shortcut learning,” where the algorithm latches onto non-biological artifacts (e.g., sample processing time) instead of disease pathology. Furthermore, the scarcity of longitudinal metabolomic data limits the ability of IML to capture dynamic disease trajectories. To ensure clinical utility, future research must pivot towards Harmonization-First approaches, prioritizing the construction of large-scale, diverse metabolic atlases. Rigorous external validation across heterogeneous populations and distinct mass spectrometry platforms is non-negotiable to prove that IML-derived biomarkers are biological facts, not statistical artifacts.

### 5.2. Interpretability Versus Clinical Trust

A fundamental paradox in current metabolomics IML is the “Multicollinearity Trap.” Biological metabolites function in tightly regulated pathways, inherently violating the feature independence assumptions of perturbation-based methods like SHAP and LIME. Consequently, these tools often suffer from attribution instability, arbitrarily splitting importance scores among co-regulated metabolites. This results in explanations that are mathematically convenient but biologically misleading—highlighting a “passenger” metabolite simply because it correlates with a “driver.” This gap between statistical importance and mechanistic causality severely erodes clinical trust; clinicians cannot rely on a “black box” that flags a metabolite without a clear, causal pathway context. Future directions must integrate Causal Structure Learning directly into IML frameworks. By constraining interpretation methods with prior biological knowledge (e.g., KEGG pathway topology), we can transition from identifying “correlated surrogates” to pinpointing causal mechanisms, thereby generating explanations that align with physiological reality.

### 5.3. Computational and Privacy Constraints

While identifying pairwise interactions is feasible, deciphering high-order metabolic interactions (e.g., three-way synergy between lipids, glucose, and insulin) is computationally prohibitive in high-dimensional spaces. Calculating exact Shapley interaction indices for thousands of features is NP-hard, forcing researchers to rely on approximation algorithms that may compromise the precision of mechanistic insights. Moreover, metabolic profiles act as unique “molecular fingerprints,” raising significant privacy concerns that restrict the sharing of raw data required to train robust deep learning models. To overcome these barriers, cutting-edge technologies like Swarm Learning (SL) offer a transformative solution. Unlike standard federated approaches, SL employs blockchain technology to collaboratively train IML models across institutions without a central coordinator or sharing raw patient spectra, thoroughly circumventing privacy silos. Simultaneously, integrating Retrieval-Augmented Generation (RAG) with LLMs serves as a concrete solution for interpretation. By anchoring high-dimensional interaction tensors directly to real-time biomedical literature and KEGG databases, RAG-enabled LLMs can automatically translate complex mathematical outputs into coherent, biologically actionable narratives, significantly reducing the cognitive load on researchers.

Beyond the currently applied algorithms, the future of precision metabolomics lies in adapting cutting-edge ML architectures that have revolutionized other domains but remain largely untested on metabolomic data. For instance, Self-Supervised Learning (SSL) and Foundation Models, which excel in natural language processing and genomics, hold immense untapped potential for untargeted metabolomics. These models could leverage vast amounts of unannotated spectral data to learn universal metabolic representations before fine-tuning for specific clinical tasks, thereby mitigating the bottleneck of metabolite annotation. Furthermore, Physics-Informed Neural Networks (PINNs), which embed known differential equations into the loss function, could be adapted into ‘Biochemical-Informed Neural Networks.’ By hardcoding known stoichiometric constraints and enzyme kinetics directly into the ML architecture, these untested methods could guarantee that model outputs strictly obey biological laws, offering a definitive leap from correlative IML to inherently mechanistic AI.

## 6. Conclusions

The integration of IML transforms metabolomics from opaque modeling toward transparent biological discovery, capturing complex, non-linear metabolic interactions. While IML successfully elucidates challenges like tumor heterogeneity, its attributions currently remain correlative. Thus, a rigorous “Dry-Wet Loop” is essential for mechanistic validation. To realize IML’s full clinical potential, future research must address specific technical pathways. Establishing standardized evaluation metrics is imperative to ensure cross-study reproducibility. The field must transcend correlation by integrating IML with causal discovery algorithms (e.g., Bayesian networks) to uncover true biological causality. Developing privacy-preserving federated modeling is vital to safely scale IML across diverse, global multi-omics cohorts. By advancing these frontiers, IML will become a foundational driver of precision metabolomics, translating high-dimensional data into robust, clinically actionable insights.

## Figures and Tables

**Table 1:** Comparison of interpretable machine learning methods in metabolomics.

Method Category	Method Name	Mechanism of Interpretation	Scope of Interpretation	Key Advantages in Metabolomics	Limitations	Use Case	References
Intrinsic Models	PLS-DA	VIP Scores (Projection-based)	Global	Handles multicollinearity. Robust for small samples (n ≪ p)	Linear relationships only. High risk of overfitting	Initial biomarker screening; discriminative profiling in untargeted MS/NMR data.	[3,30]
	Lasso/Elastic Net	Regression Coefficients (β)	Global	Lasso: Sparse feature selection. Elastic Net: Retains grouped correlated metabolites	Assumes linearity. Lasso may drop highly correlated redundant features	Identifying sparse, clinically translatable biomarker panels from high-dimensional datasets.	[31,32,33]
	Decision Trees	Split Nodes & Gini Impurity	Global/Local path	Intuitive “If-Then” logic. Explicit threshold visualization	Highly unstable to noise. Lower predictive accuracy vs. ensembles	Deriving simple, tree-based clinical diagnostic cut-offs for targeted metabolite assays.	[34,35]
	GAMs	Shape Functions	Global	Models’ non-linear effects natively. Maintains strict additivity	Computationally heavy for large p. Misses complex feature interactions	Mapping non-linear dose–response relationships (e.g., U-shaped metabolic toxicity curves).	[36,37]
	PCA	Loadings (Eigenvectors)	Global	Unbiased baseline visualization. Reveals natural data structure	Unsupervised (ignores class labels). Focuses on high variance, not discrimination	Exploratory data analysis; detecting batch effects and outliers in raw quality control (QC) samples.	[1,3]
Post hoc Model-Agnostic	SHAP	Shapley Values (Game Theory)	Global & Local	Theoretically consistent (axiomatic). Unifies global, local, & interaction analysis	Extremely computationally intensive. Often assumes feature independence	Uncovering deep biological mechanisms; evaluating metabolite-metabolite synergistic or antagonistic interactions.	[11,20]
	LIME	Local Linear Approximation	Local	Highly model-agnostic. Easy to implement for individual predictions	Highly unstable to sampling variations. Lacks global dataset perspective	Explaining individual patient misclassifications; personalized single-sample metabolic anomaly detection.	[6,38]
	PFI	Permutation-based Error Increase	Global	Computationally fast. Highly intuitive & model-agnostic	Severely biased by multicollinearity. Fails to capture feature interactions	Rapid, preliminary global ranking of significant biomarkers in strictly uncorrelated metabolic panels.	[39,40]
	PDP/ALE Plots	PDP: Average Marginal Effect; ALE: Accumulated Local Effects	Global	Visualizes non-linear functional forms. ALE natively handles severe multicollinearity	PDP generates unrealistic synthetic data points. ALE can be non-intuitive for clinicians	Identifying non-linear metabolic thresholds (e.g., toxicity tipping points, enzyme saturation, or biological “Goldilocks zones”).	[21,41]
	Counterfactuals	Minimum Perturbation Analysis	Local	Generates highly actionable “what-if” scenarios. Intuitively aligns with clinical decision-making	Optimization is computationally intractable. Suffers from the “Rashomon effect” (multiple valid solutions)	Formulating personalized therapeutic interventions (e.g., guiding specific dietary modifications or targeted metabolite supplementation).	[23,42]
Deep Learn-ing-Specific	LRP	Relevance Propagation	Local (Pixel/Feature)	Feature-level decomposition for spectral data	Complex implementation. Restricted to neural networks.	Identifying key diagnostic spectral peaks in raw NMR/MS data for targeted biomarker extraction.	[43,44]
	Integrated Gradients	Path Integral of Gradients	Local	Overcomes gradient saturation. Mathematically complete.	Computationally heavy (requires multiple forward passes).	Precise metabolite feature attribution in complex deep multi-omics networks (e.g., CNN/DNN).	[24,45]
	Attention	Attention Weights	Local/Global	Intrinsic interpretability for nodes and pathways.	Restricted to specific architectures (Transformers/GNNs).	Elucidating metabolic pathway interactions and topological importance using Graph Neural Networks.	[25,26]
	Grad-CAM	Gradient-weighted Activation Maps	Local	Visualizes specific model activation regions.	Coarse resolution; strictly limited to CNN architectures.	Spatially locating discriminative metabolite regions directly from 2D NMR/MS spectral images.	[4]
Feature Selection Wrappers	Boruta	Shadow feature comparison (Random Forest)	Global	“All-relevant” selection of biologically linked metabolites	High computational cost. Tied to Random Forest architecture.	Exploratory biomarker discovery mapping comprehensive, highly correlated metabolic pathways.	[27]
	RFE	Iterative pruning based on weights	Global	“Minimal-optimal” selection. Maximizes accuracy with fewest features.	Greedy algorithm Sensitive to multicollinearity (drops correlated features).	Developing streamlined, cost-effective clinical diagnostic panels using essential metabolic subsets.	[22]

**Table 2:** Selected case studies demonstrating IML-driven insights in metabolomic biomarker discovery.

Disease Context	Clinical Challenge	IML Methodology	Differential Metabolites	Key Metabolic Insight	Mechanistic/Clinical Implication	References
Alzheimer’s Disease (AD)	Detecting early-stage (MCI) signals obscured by biological noise and non-linearity.	Deep Learning + Integrated Gradients (IG)	Phosphatidylcholines (PC), Lysophosphatidylcholines (LPC), Sphingomyelins (SM), PC/LPC Ratios	Uncovered non-linear predictive power of lipid ratios, outperforming linear models.	Reflects membrane instability/PLA2 hyperactivity; targets lipid remodeling for early intervention.	[20]
Breast Cancer	Distinguishing subtypes (TNBC vs. Non-TNBC) beyond receptor status.	XGBoost + SHAP (Global & Local Explanations)	Phosphatidylcholines (e.g., PC aa C36:2), Choline, Phosphocholine, Glycerophosphocholine	SHAP revealed lipid saturation patterns driving TNBC classification beyond critical thresholds.	Links Choline upregulation to the aggressive, proliferative phenotype of TNBC tumors.	[55]
Colorectal Cancer	Integrating microbiome-metabolome interactions for non-invasive screening.	Random Forest + Feature Importance & Correlation Network	Desaminotyrosine (DAT), Flavonoid metabolites, Short-Chain Fatty Acids (SCFAs), Secondary Bile Acids	Identified synergistic metabolite-microbiome interactions and host-microbial co-dependency.	Validates microbial-immune modulation via flavonoids; enhances specificity vs. FOBT.	[58]
Type 2 Diabetes (T2DM)	Predicting onset years before clinical diagnosis using complex interactions.	Gradient Boosting (CatBoost) + SHAP	Branched-Chain Amino Acids (Leucine, Isoleucine, Valine), Aromatic Amino Acids (Tyrosine, Phenylalanine)	Visualized non-linear risk thresholds and concentration tipping points accelerating disease probability.	Defines pre-diabetic window; guides dietary interventions via BCAA thresholds.	[32]
Endometrial Cancer	Reducing false positives in serum biomarker screening.	Machine Learning Ensemble + SHAP Dependence	Stearamide, Hypoxanthine, Phospholipids (PC, PE), Xanthine, Inosine	SHAP plots visualized precise non-linear contributions of lipid and purine markers.	Suggests Purine/lipid dysregulation; complements ultrasound via liquid biopsy.	[34]
Depression (MDD)	Identifying metabolic subtypes of depression.	Logistic Regression/SVM + RFE	Tryptophan, Kynurenine, Kynurenic Acid, Quinolinic Acid, Serotonin	Validated a multi-metabolite panel distinguishing MDD via pathway-level dysregulation.	Links Neuroinflammation to symptoms; supports anti-inflammatory psychiatric therapies.	[78]
Pancreatic Cancer (PDAC)	Differentiating PDAC from benign pancreatic disease.	Support Vector Machine + Feature Selection	Glutamine, Glutamate, Proline, Lysine, Histidine, Citrulline, Sphingomyelin	Established robust signature distinguishing PDAC from pancreatitis.	Distinguishes tumor metabolic reprogramming from inflammation.	[79]
COVID-19 Severity	Predicting progression to severe respiratory failure.	Random Forest + Boruta	Kynurenine, Tryptophan, Creatinine, Triglycerides, Phenylalanine	Revealed “lock-step” amino acid and lipid correlations tracking disease severity.	Indicates immune exhaustion, mitochondrial dysfunction; guides metabolic support.	[80]
Cardiovascular Disease	Predicting heart failure risk in a heterogeneous population.	XGBoost + SHAP Interaction Values	Long-chain Acylcarnitines, β-Hydroxybutyrate (Ketone bodies), Acetone, Fatty Acids	Discovered high-order substrate interactions undetectable by conventional linear models.	Identifies Metabolic Inflexibility as core pathology; refines diabetic risk stratification.	[81]
Chronic Kidney Disease	Identifying toxins driving uremic symptoms.	Linear Models + Empirical Bayes (High-Dim)	Hippurate, Indoxyl Sulfate, p-Cresyl Sulfate, Phenylacetylglutamine, Uremic Toxins	Linked uremic solute accumulation to neurocognitive symptom clusters via multivariate analysis.	Demonstrates Gut-Kidney crosstalk; supports targeting gut-derived toxins for symptom relief.	[82]
Aging (Biological Age)	Distinguishing biological from chronological age.	Elastic Net Regression	Glutamine, Carnitines, Specific Lipid species (SM, PC), Steroid Hormones	Constructed a metabolomic score predicting healthspan superior to chronological age.	Proposes a “Metabolic Aging Clock”; guides lipid-targeted anti-aging interventions.	[83]
NAFLD/NASH	Non-invasive diagnosis of steatohepatitis.	Random Forest + Recursive Partitioning	Glutamate, Isocitrate, Bile Acids (Glycocholic acid), Taurine	Developed “MetaNASH” score stratifying progressive steatohepatitis from benign steatosis.	Reflects mitochondrial dysfunction/oxidative stress; replaces invasive liver biopsy.	[84]
Ovarian Cancer	Pre-operative diagnosis via liquid biopsy.	Machine Learning (SVM/RF) + Feature Selection	Histidine, Tryptophan, Citrulline, Hydroxyphenyllactic acid, Specific Phospholipids	Discriminated early-stage malignancy from benign cysts via distinct amino acid/lipid shifts.	Confirms Warburg-like metabolic shifts; facilitates early patient triage.	[85]
Gestational Diabetes (GDM)	Early prediction in the first trimester.	Logistic Regression (Lasso) + Nomogram	3-Hydroxybutyrate, Alanine, Valine, Proline, Hexose	Visualized individual risk probabilities via a compact, clinically viable nomogram.	Signals early Insulin Resistance; guides diet before hyperglycemia.	[86]
Toxicology (Drug Safety)	Detecting organ-specific toxicity signatures.	AutoML (XGBoost) + SHAP Summary	Taurine, Hippurate, Citrate, Bile Acids, Creatine	SHAP flagged subtle perturbations as early warning signals for liver injury (DILI).	Links xenobiotic metabolism to oxidative stress; provides pharma safety screening tool.	[41]

## Data Availability

No new data were created or analyzed in this study. Data sharing is not applicable.
